# Social Media Use, eHealth Literacy, Knowledge, Attitudes, and Practices Toward COVID-19 Vaccination Among Chinese College Students in the Phase of Regular Epidemic Prevention and Control: A Cross-Sectional Survey

**DOI:** 10.3389/fpubh.2021.754904

**Published:** 2022-01-27

**Authors:** Ning Qin, Shuangjiao Shi, Yinglong Duan, Guiyue Ma, Xiao Li, Zhiying Shen, Shuhua Zhang, Aijing Luo, Zhuqing Zhong

**Affiliations:** ^1^Nursing Department, Third Xiangya Hospital, Central South University, Changsha, China; ^2^Department of Cardiology, The Third Xiangya Hospital of Central South University, Changsha, China; ^3^Department of Emergency, The Third Xiangya Hospital of Central South University, Changsha, China; ^4^Xiangya School of Nursing, Central South University, Changsha, China; ^5^Key Laboratory of Medical Information Research, College of Hunan Province, Central South University, Changsha, China

**Keywords:** COVID-19 vaccination, social media, media use, eHealth literacy, KAP (knowledge, attitudes, and practices)

## Abstract

**Background:**

College students are at a high risk of being infected with COVID-19, and they are one of the key population clusters that should be vaccinated. The present study aimed to investigate the knowledge, attitudes, and practices (KAP) toward COVID-19 vaccination among Chinese college students, and to determine the relationships among social media use, eHealth literacy, and KAP toward COVID-19 vaccination among Chinese college students.

**Methods:**

A cross-sectional survey was conducted by administering questionnaires to evaluate KAP toward COVID-19 vaccination, social media use, and eHealth literacy in one of the groups of Chinese college students. Multiple linear regression analysis was performed to determine the association among social media use, eHealth literacy, and KAP regarding COVID-19 vaccination.

**Results:**

Among the 3,785 validated questionnaires collected from Chinese college students, male students accounted for 59.74%, and the mean age of the college students was (20.90 ± 3.14) years. More than four-fifths (83.43%) of the college students spent <2 h a week on social media, and the official and public social media were most common social media types. Additionally, the scores for KAP toward COVID-19 vaccination ranging from 0 to 48 among college students were high (39.73 ± 5.58), lowest for knowledge domain (3.07 ± 0.76), and the highest for practice domain (3.47 ± 0.63). Female college students who were in good health status and who spent more time browsing social media, frequently used official and public social media, rarely used aggregated social media, and had a relatively strong self-perception of eHealth literacy and information acquisition of eHealth literacy were more likely to have high levels of KAP regarding COVID-19 vaccination.

**Conclusions:**

Overall, Chinese college students have excellent KAP toward COVID-19 vaccination. Based on the findings of this study, we recommend that health counseling regarding COVID-19 vaccination should target male students and those with inferior health status. Dissemination of health education regarding COVID-19 vaccination should be purposely conducted, and cooperation with official and public social media platforms should be promoted. Finally, eHealth literacy, which is one of the predictors of the level of KAP regarding COVID-19 vaccination, should be emphasized.

## Introduction

### Background

COVID-19 outbreak emerged at the end of 2019 and was declared a pandemic by the World Health Organization (WHO) in early 2020. COVID-19 has rapidly spread across the globe in a short period of time, and it is characterized by high infectivity, long incubation period, and asymptomatic infection. WHO proclaimed that qualified COVID-19 vaccines sanctioned by governments could be highly protective against infection with COVID-19 (1). The vaccines can effectively protect populations against infection with COVID-19 and severe disease symptoms such as, severe pneumonia, in turn, reducing mortality associated with COVID-19. Therefore, individuals have been urged to go in for vaccinations as soon as possible. By June 01, 2021, a total of 158 million doses of vaccines had been administered across the globe. The rates of COVID-19 vaccination in America and Canada are over 50%; 20% in Brazil, Argentina, and China; and 10% in Russia and India. The attitudes of people from different regions toward COVID-19 vaccination are varied. One global research conducted recently revealed that 71.5% of the people were willing to receive COVID-19 vaccines, and willingness among the Chinese population was the highest (88.6%), whereas willingness among the Russian population was the lowest (54.9%) (2).

Studies have revealed that the willingness of individuals to get vaccinated has been on a downward trend since the spread of COVID-19 pandemic was contained (3). Specifically, females, young adults, individuals with low education level or with low socio-economic status, and people from minority ethnic groups were more likely to reject COVID-19 vaccination. College students are well-educated young adults and they are at high risk of exposure to and infection with COVID-19 due to their active social lives and great social mobility. Therefore, college students are one of the key populations that require vaccination. However, previous studies have revealed that the willingness of college students to accept COVID-19 vaccination is not satisfactory. The proportion of American college students who are hesitant to get vaccinated is 47.5% (4); among them, 45.0% are dental students and 23.0% are medical students (5), and the case is similar to that of students in Jordan University (6). Sun et al. (7) investigated the attitude of Chinese students toward COVID-19 vaccines during the COVID-19 pandemic, and the results revealed that 64.01% of the college students were willing to take shots of COVID-19 vaccines as part of the vaccination trials. Students with low socio-economic status, women, and individuals with a stronger self-perception of susceptibility to COVID-19 were more likely to show willingness to take COVID-19 vaccines. However, in the present situation of regular COVID-19 prevention and control, KAP regarding COVID-19 vaccination among Chinese college students remains unknown.

During the period of COVID-19 pandemic, social media use may have substantial impacts on the attitudes and willingness of people to accept COVID-19 vaccination. A study conducted by Puri et al. revealed that individuals who used new media to acquire information regarding the pandemic were more likely to be hesitant to receive COVID-19 vaccination than those who relied on traditional media (8). Sallam et al. established that reliance on social media for COVID-19-related information instead of doctors, authoritative specialists, and scientific periodicals was significantly associated with less willingness to receive COVID-19 vaccination (6). Contrary to new media platforms, Romer and Jamieson found that mainstream broadcast TV had a positive effect on facilitating vaccination willingness among American residents (9). Allington et al. pointed out that as a major source of information among British residents, social media platforms had a positive association with belief in a specific form of misinformation. In addition, a negative association was observed between health-protective behaviors against COVID-19 and belief in a specific form of misinformation (10). A study by Li and Liu revealed that the use of aggregated social media was identified as the optimal predictor of health-protective behaviors among Chinese residents, whereas the use of official social media was not significantly associated with health-protective behaviors among the Chinese population (11). The results of the Chinese population were not consistent with the findings of previous studies. Generally, further research is required to determine whether the use of social media is associated with KAP toward COVID-19 vaccination among Chinese students, as well as the effects of social media use on KAP toward COVID-19.

eHealth literacy is the ability to search, find, understand, and make critical evaluation of online health information from electronic health resources to address health problems (12). A much closer association was observed between health-protective behaviors against COVID-19 and eHealth literacy than with health literacy (13). A high level of eHealth literacy tends to prompt health-protective behaviors. A previous study revealed that nursing students with relatively high levels of eHealth literacy were inclined to engage in more positive protection practices against COVID-19 (14). Specifically, a combination of different types of eHealth literacy enhances the adoption of health-protective behaviors. A study by Yang et al. divided eHealth literacy into three categories: functional eHealth literacy, interactive eHealth literacy, and critical eHealth literacy. The study revealed that students with high levels of interactive and critical eHealth literacy were more likely to embrace positive and healthy diets, whereas students with only high levels of interactive eHealth literacy were subject to misinformation and tended to adopt unbalanced diets (15). eHealth literacy was identified as one of the crucial predictors of heath-protective behaviors among Chinese populations during the COVID-19 pandemic period (11). The association between eHealth literacy and KAP toward COVID-19 vaccination among Chinese college students remains unknown. Moreover, the effects of the different types of eHealth literacy on KAP toward COVID-19 vaccination among Chinese college students require further investigation.

Based on the aforementioned background, the present study aimed (1) to investigate KAP toward COVID-19 vaccination, use of social media, and eHealth literacy among Chinese college students and (2) to identify the association between KAP toward COVID-19 vaccination and demographic characteristics, use of social media, and eHealth literacy. The results of this study could provide a basis for further research on promoting KAP toward COVID-19 vaccination among Chinese college students. The results could also provide a reference for disseminating and managing health information regarding COVID-19 vaccination from the perspective of social media use and eHealth literacy.

### Hypotheses

We proposed the following three hypotheses with respect to the association between KAP toward COVID-19 vaccination and demographic characteristics, social media use, and eHealth literacy.

#### Hypothesis 1

Demographic characteristics are associated with KAP toward COVID-19 vaccination among Chinese college students (11, 16–19).

1a: KAP toward COVID-19 vaccination is associated with gender, age, health status, majors, and education level. Specifically, female students who were older, majored in medical subjects, and had high education levels were more likely to have high levels of KAP toward COVID-19 vaccination.

1b: KAP toward COVID-19 vaccination is correlated with earlier infection of people around the students with COVID-19 including relatives, friends, and classmates, which predicts high levels of KAP toward COVID-19 vaccination.

1c: KAP toward COVID-19 vaccination is associated with self-perception of susceptibility to COVID-19. A stronger self-perception of susceptibility predicts high levels of KAP toward COVID-19 vaccination.

#### Hypothesis 2

Social media use is associated with KAP toward COVID-19 vaccination (11, 18, 20).

2a: Weekly time spent browsing social media is positively correlated with KAP toward COVID-19 vaccination.

2b: Weekly frequency of using social media is positively correlated with KAP toward COVID-19 vaccination.

2c: The use of different types of social media platforms is a key predicting factor of different levels of KAP toward COVID-19 vaccination.

#### Hypothesis 3

eHealth literacy is associated with KAP toward COVID-19 vaccination (11, 17, 21).

3a: Level of eHealth literacy is positively correlated with the level of KAP toward COVID-19 vaccination.

3b: Various domains within eHealth literacy are key predicting factors of different levels of KAP toward COVID-19 vaccination.

## Materials and Methods

### Research Design and Recruitment of Participants

The present study was a cross-sectional investigation and was conducted at a university in Changsha city, Hunan province of China, which is one of the prestigious universities in China. The university offers bachelor's degree programs in 106 majors and has over 50,000 full-time students. Questionnaires were made accessible through a QR code using an online platform, “Questionnaire Star” (URL: https://www.wjx.cn/). The students scanned the QR code using the WeChat scanning function to access the specific questionnaires. Every single WeChat ID was allowed to complete the questionnaires only once. In this study, participants were reached both online through WeChat groups and onsite in the campus. Approvals were obtained from form masters of the university and the QR code was sent to the WeChat groups for students undertaking various majors. Participants were also reached during their break times while in the campus. Students who agreed to participate in the survey signed informed consent forms. The survey was conducted from May 1, 2021 to May 23, 2021. The research background, objectives, principle of anonymity and confidentiality, notes for attention, and agreement statements to fill in the questionnaires were explained to the participants before they completed the questionnaires. The inclusion criteria were as follows: (1) students aged over 18 years; (2) Chinese students from the selected university; (3) students who agreed to participate in the survey.

### Study Measures

#### Demographic Information

Demographic characteristics included gender, age, major, educational level, health status, whether the relatives, friends, or classmates have been infected with COVID-19, and their self-perception of susceptibility.

#### Social Media Use

Social media use was evaluated based on two aspects, and according to a previous study, they are: time spent browsing social media and the frequency of using different types of social media platforms (11). For evaluating the browsing time, we used this question in our study, “How much time did you spend on social media searching for information regarding COVID-19 over the past week?”; it had the following response options “ <1 h a week, 1–2 h a week, 2–3 h a week, 3–4 h a week, 4–5 h a week, and over 5 h a week,” and we scored each option with 1–6, respectively. For evaluating the frequencies for using different types of social media, we use this question “Which social media platform and how often do you use it to get online information about the COVID-19?” in our study. Participants responded with “never (scoring 1),” “1–2 times a week (scoring 2),” “3–4 times a week (scoring 3),” “5–6 times a week (scoring 4),” and “once a day or more often than that (scoring 5).” Social media were classified into four categories: (1) official social media (such as CCTV news and The People's Daily); (2) medical professional social media (such as “Doctors Clove” and “Micro Medicine”); (3) public social media (such as WeChat, MicroBlog, and Chinese version of TikTok); (4) aggregated social media (such as “Tencent News,” “Tou Tiao,” and “Wang Yi News”). The total score with a range of 4–20 was calculated by adding individual frequencies of each type of social media, and higher scores indicated higher frequencies of using social media.

#### eHealth Literacy

eHealth literacy among Chinese college students was evaluated using the mobile eHealth Literacy Scale (m-eHEALS), which included 12 items within 3 domains: self-perception (3 items), information acquisition (5 items), and interactive evaluation (4 items). The responses for each question on the scale were as follows: strongly disagree (scoring 1), disagree (scoring 2), not sure (scoring 3), agree (scoring 4), and strongly agree (scoring 5). The total score for the entire scale ranged from 12 to 60, and higher scores indicated higher levels of eHealth literacy. The m-eHEALS was first developed in 2017 to evaluate eHealth literacy among customers with regard to the prevailing online consumption of medical products (21). The Cronbach α coefficient for the scale in the present study population was 0.942.

#### KAP Toward COVID-19 Vaccination

Questionnaires for evaluating KAP toward COVID-19 vaccination were developed by a research team based on the theoretical model of KAP. The questionnaire included 12 items within 3 domains of knowledge (4 items), attitudes (4 items), and practices (4 items).

The items in the knowledge domain were developed based on the guidelines for COVID-19 vaccination (version one) (22), and the questions and answers regarding COVID-19 vaccination (23) issued by the National Health Council and the Centers for Disease Control and Prevention. All items in the knowledge domain had the following response categories: true, false, or don't know. Correct answers scored 4, whereas incorrect answers or choosing “don't know” scored 0. Items 1 and 3 scored reversely. Items in the attitude domain were developed based on the theory of psychological antecedents of vaccination (24). In addition, participants were evaluated in terms of vaccination confidence, risk assessment, self-control, and collective responsibility. The practices for COVID-19 vaccination were assessed based on the health information seeking behaviors regarding COVID-19 vaccines, practices associated with information sharing, practices associated with taking shots of COVID-19 vaccines, and protective behavior after vaccination. Response options for items in the attitude and practice domains were: strongly disagree, disagree, not sure, agree, strongly agree, which were scored as 0, 1, 2, 3, and 4, respectively. Item 4 in the attitude domain scored reversely. The total score for the KAP toward COVID-19 vaccination questionnaire ranged from 0 to 48, and higher scores indicated higher levels of KAP toward COVID-19 vaccination. The Cronbach α coefficient for the measures was 0.744. The survey instruments can be found in Appendix 1 in the Supplementary Material.

#### Data Analysis

IBM SPSS Statistics 25.0 (IBM Corporation, USA) was used for statistical analyses. With regard to descriptive analyses, categorical variables, including gender, age, major, education level, health status, previous infection of close contacts with COVID-19, and self-perception of susceptibility to COVID-19, were presented as frequencies and percentages. Continuous variables, such as daily or weekly time spent on social media, frequencies of using different types of social media platforms, the total frequencies of using various types of social media platforms, the level of eHealth literacy, and the level of KAP toward COVID-19 vaccination, were presented as means ± standard deviation. Univariate analysis was performed using independent *t*-test or one-way analysis of variance. Pearson's correlation analyses were performed to determine correlations among social media use, eHealth literacy, and KAP toward COVID-19 vaccination. Multiple linear regression analysis was performed to identify the determinants of KAP. A variance inflation factor (VIF) was used to determine multicollinearity among variables. A *p* < 0.05 in a two-tailed test was considered statistically significant.

## Results

### Socio-Demographic Characteristics

A total of 4,629 eligible college students completed the questionnaires. Invalidated responses were screened out as follows: a total of 420 responses were discarded because the time taken to complete the questionnaires was <2 min (2 min was considered the minimum time needed to complete the questionnaires during the pilot survey); a total of 424 responses were deleted due to consistent responses for different questions. We finally included 3,785 responses in our statistical analyses. Validated response rate of the survey was 81.77%.

The results revealed that among the 3,785 college students who participated in the present survey, male students accounted for 59.74% and female for 40.26%. The mean age of the study participants was (20.90 ± 3.14) years, and the majority were non-medical students (85.84%) and undergraduate students (75.75%). The proportion of students with very good or pretty good health status was 98.26%. A few of the students (0.42%) had relatives, friends, or classmates who had been infected with COVID-19 (Table 1). Furthermore, more than half of the college students (66.84%) thought that they were not likely to be infected with COVID-19.

**Table 1:** Socio-demographic characteristics and univariate analysis for KAP toward COVID-19 vaccination.

**Variables**	***N*** **(%)**	**The score for KAP toward COVID-19 vaccination (M ±SD)**	***t/F***	***P***
**Gender**			−3.96	<0.001
Male	2,261 (59.74)	39.44 ± 5.79		
Female	1,524 (40.26)	40.16 ± 5.23		
**Age (years)**			3.33	0.019
18–20	2,173 (57.41)	39.79 ± 5.65		
21–23	981 (25.92)	39.31 ± 5.54		
24–26	441 (11.65)	40.19 ± 5.35		
≥27	190 (5.02)	40.16 ± 5.41		
**Major**			3.07	0.002
Medical	536 (14.16)	40.42 ± 5.28		
Non-Medical	3,249 (85.84)	39.62 ± 5.62		
**Education**			1.43	0.240
Undergraduate	2,867 (75.75)	39.67 ± 5.66		
Post-Graduate	728 (19.23)	39.80 ± 5.28		
PhD	190 (5.02)	40.36 ± 5.44		
**Health status**			23.71	<0.001
Good	3,129 (82.67)	40.00 ± 5.56		
Pretty good	590 (15.59)	38.65 ± 5.47		
Average level, fairly poor, poor	66 (1.74)	36.86 ± 4.68		
**Whether surrounding relatives, friends, or classmates ever infected with COVID-19**			−0.57	0.568
Yes	16 (0.42)	38.94 ± 5.43		
No	3769 (99.58)	39.74 ± 5.58		
**Self-perception of the possibility of being infected with COVID-19**			3.57	0.013
Impossible	793 (20.95)	40.15 ± 5.67		
Not likely	2530 (66.84)	39.56 ± 5.55		
Possible	455 (12.02)	39.88 ± 5.58		
Most likely or certainly	7 (0.18)	43.71 ± 1.80		

*t, Two-sample t-test; F, one-way analysis of variance*.

### Social Media Use, eHealth Literacy, and KAP Toward COVID-19 Vaccination

The results revealed that 83.43% of the college students spent <2 h a week on social media, and only 3.78% of the college students spent more than 5 h on social media (the detail information can be found in Appendix 2 in the Supplementary Material). The mean score for weekly time the college students spent on social media was (1.82 ± 1.19). The total score for weekly frequencies in using various types of social media platforms was (8.89 ± 3.18). Chinese college students were more inclined to browse official and public social media for information regarding COVID-19 and vaccination. Conversely, aggregated and medical professional social media were not frequently used. The mean score for eHealth literacy was (46.61 ± 8.16). The domains of eHealth literacy were scored in the order of information acquisition > self-perception > interactive judgment. The total score of KAP toward COVID-19 vaccination was (39.73 ± 5.58), and the mean scores of the three domains in descending order were as follows: knowledge > attitudes > practices. See detailed scores in Table 2.

**Table 2:** Social media use, eHealth literacy, and KAP toward COVID-19 vaccination.

**Variables**	**M ±SD**
The score for weekly time spent on social media	1.82 ± 1.19
The total score for weekly frequencies in using various types of social media platforms	8.89 ± 3.18
The mean score for weekly frequencies in using official social media	2.54 ± 1.10
The mean score for weekly frequencies in using medical professional social media	1.71 ± 1.00
The mean score for weekly frequencies in using the public social media	2.54 ± 1.17
The mean score for weekly frequencies in using aggregated social media	2.10 ± 1.08
The total score for eHealth literacy	46.61 ± 8.16
The mean score for self-perception domain	3.90 ± 0.75
The mean score for information acquisition domain	4.02 ± 0.69
The mean score for interactive judgment domain	3.70 ± 0.80
The total score for KAP toward COVID-19 vaccination	39.73 ± 5.58
The mean score for knowledge domain	3.07 ± 0.76
The mean score for attitude domain	3.39 ± 0.60
The mean score for practice domain	3.47 ± 0.63

*M, Mean; SD, standard deviation*.

### Factors Influencing KAP Toward COVID-19 Vaccination

The results of univariate analyses revealed that female students, students aged over 24 years, students who majored in medical subjects, students with superior health status, and students with a stronger self-perception of susceptibility to COVID-19 had significantly higher scores of KAP toward COVID-19 vaccination (*p* < 0.05).

Weekly time spent on social media, total frequencies of using various types of social media, and total score for eHealth literacy were positively correlated with the total score for KAP toward COVID-19 vaccination (*r* = 0.117, *r* = 0.168, *r* = 0.377, *p* < 0.001). Specifically, college students who spent longer time on social media had higher frequencies of using various types of social media, higher levels of eHealth literacy, and higher scores of KAP toward COVID-19 vaccination. In addition, attitude and practice of KAP toward COVID-19 vaccination were positively correlated with eHealth literacy and each of its domains, as well as weekly frequencies of using various types of social media. In summary, the results supported hypotheses 2a, 2b, and 3a. See details in Table 3.

**Table 3:** The correlation analysis for social media use, eHealth literacy, and KAP toward COVID-19 vaccination.

**Variables**	**Total score for KAP toward COVID-19 vaccination**	**knowledge**	**attitudes**	**practices**
Weekly time spent on social media	0.117^*^	0.023	0.133^*^	0.104^*^
Frequencies in using varying social media	0.168^*^	0.017	0.144^*^	0.215^*^
Official social media	0.172^*^	0.023	0.154^*^	0.208^*^
Medical professional social media	0.097^*^	0.033^*^	0.062^*^	0.116^*^
Public social media	0.132^*^	0.008	0.122^*^	0.167^*^
Aggregated social media	0.086^*^	−0.013	0.078^*^	0.133^*^
Total score for eHealth literacy	0.377^*^	0.007	0.382^*^	0.467^*^
Self-Perception	0.358^*^	0.000	0.377^*^	0.437^*^
Information acquisition	0.383^*^	0.014	0.391^*^	0.462^*^
Interactive judgment	0.297^*^	0.002	0.287^*^	0.384^*^

* *P < 0.001*.

Multiple linear regression analysis was conducted with KAP toward COVID-19 vaccination as the dependent variable and it included independent variables with socio-demographic characteristics that were initially identified to be significant in the univariate analysis, social media use, and also the three domains of eHealth literacy that had been previously demonstrated to be significantly correlated with KAP toward COVID-19 vaccination (α _in_ = 0.05, α _out_ = 0.10). The socio-demographic characteristics were gender (1 = male; 2 = female), age (1 = 18–20 years; 2 = 21–23 years; 3 = 24–26 years; 4 = ≥ 27 years), major (1 = medical subjects; 2 = nonmedical subjects), health status (1 = very good; 2 = pretty good; 3 = average level, fairly poor, or poor), self-perception of the possibility of being infected with COVID-19 (1 = impossible; 2 = not likely; 3 = possible; 4 = most likely or certainly). The results of multiple linear regression analysis are presented in Table 4.

**Table 4:** Multiple linear regression analysis on KAP toward COVID-19 vaccination among Chinese college students (*N* = 3,785).

**Variables**	**Partial regression coefficient**	**Standard error**	**Standardized partial regression coefficient**	**t**	***P***
Constant	26.656	0.866	–	30.774	<0.001
Gender	0.716	0.174	0.063	4.125	<0.001
Health status	−0.740	0.193	−0.058	−3.828	<0.001
Time spent on social media use	0.208	0.073	0.044	2.845	0.004
Frequencies in using official social media	0.508	0.089	0.100	5.732	<0.001
Frequencies in using professional medical social media	−0.127	0.094	−0.023	−1.356	0.175
Frequencies in using the public social media	0.203	0.086	0.043	2.377	0.018
Frequencies in using aggregated social	−0.207	0.092	−0.040	−2.243	0.025
Self-Perception of eHealth literacy	0.333	0.066	0.135	5.064	<0.001
Information acquisition of eHealth literacy	0.400	0.046	0.248	8.618	<0.001
Interactive judgment of eHealth literacy	0.003	0.040	0.002	0.066	0.947

*F = 62.527, P < 0.001, adjusted R^2^ = 0.174*.

The results of regression analyses revealed that VIF was 1.03–3.79, suggesting there was no multicollinearity among the included independent variables. Eight variables that were statistically significant, including gender, health status, weekly time spent on social media, frequencies of using official social media, public social media and aggregated social media, self-perception of eHealth literacy, and information acquisition of eHealth literacy were retained. Particularly, female students, students with good or pretty good health status, students who spent more time on social media, students with relatively high frequencies of using official or public social media, low frequencies of using aggregated social media, as well as students with a stronger self-perception of eHealth literacy and information acquisition of eHealth literacy were more likely to have higher levels of KAP toward COVID-19 vaccination. Therefore, the results supported hypotheses 2c and 3b, while hypotheses 1b and 1c were not supported by the results of the present study. Moreover, hypothesis 1a was partially proven to be true. Female students and students with good or pretty good health status had higher levels of KAP toward COVID-19 vaccination.

## Discussion

The findings showed that the KAP toward COVID-19 vaccination among Chinese college students was relatively high in the phase of regular epidemic prevention and control. Most college students (83.43%) spent <2 h searching for COVID-19 information. Most students mainly focused on official social media or public social media, whereas only a few explored the aggregated social media and professional medical social media. Chinese college students were equipped with good eHealth literacy. The findings showed high scores on information acquisition for college students; however, they presented low scores in the ability of interactive judgment. These findings were consistent with the findings of a study by Wu et al. (21) that the ability of interactive judgment on online health information should be emphasized and improved among Chinese college students. Notably, female students and students with good health status showed higher level of knowledge, more positive attitude, and practices to accepting shots of vaccines against COVID-19. In addition, this group spent more time using social media to search for information on COVID-19 and vaccination. Notably, the group exhibited higher frequencies in using official and the public social media, and less frequencies in using aggregated social media. Notably, stronger self-perception of eHealth literacy and information acquisition of eHealth literacy were significant factors in predicting higher level of KAP of COVID-19 vaccination. Firstly, it indicates that the official and the public social media platforms should be utilized and equipped with authoritative health information on COVID-19 and vaccination. Online channels for disseminating health information on COVID-19 and vaccination should therefore be optimized. In addition, male students, individuals with inferior health status, and individuals with lower level of eHealth literacy should be targeted to identify their demands for COVID-19 vaccination, and as a result the specific limitations to vaccination against COVID-19 among Chinese college students can be determined. Tailored interventions can be implemented to ensure 100% COVID-19 vaccination coverage in China.

### Demographics and KAP Toward COVID-19 Vaccination

The findings of the current study showed that female students and students with good health status were more likely to have higher level of KAP toward COVID-19 vaccination compared with male students. These findings were consistent with findings from previous studies. Li and Liu reported that female residents and those in good health status were more likely to undergo positive prevention measures during the COVID-19 pandemic (11). This may be due to higher level of knowledge of the COVID-19 pandemic and higher positive responding attitude among females. In addition, this observation can be attributed to stronger self-perception of susceptibility and higher self-perceived benefits of being vaccinated against COVID-19 among Chinese female residents (25). On the contrary, the findings showed that college students with inferior health status would worry about contraindications of COVID-19 vaccination and its potential harm to their health, resulting in lower vaccination rates (26). Notably, a systematic review across countries reported that the number of residents who were willing to undergo COVID-19 vaccination declined as the pandemic was contained. Moreover, the review reported the female and residents in lower income areas were more likely to decline COVID-19 vaccination (3). A limitation of the systematic review was that only 1 study conducted in China was included in the review, and the study from China was not part of the comprehensive analysis on socio-demographics. This indicates that there was discrepancy on gender impacts between China and other countries, which can be attributed to different culture backgrounds and different government policies on COVID-19 vaccination (such as free access to COVID-19 vaccination in China) (27). Furthermore, this can be attributed to the analysis of different populations for the present study and the systematic review. In addition, college students were the target population in the present study, whereas a larger proportion of pregnant women or those prepared for pregnancy were among the participants in the studies included in the systematic review, who may have been concerned with the adverse reactions caused by COVID-19 vaccines. Rozenberg et al. reported similar infection rate between the male and female population globally; however, male patients had 0.6-fold higher risk to present with critical pneumonia or with other complications (28). A survey in China reported that men with critical COVID-19 pneumonia or with other complications presented higher risk of mortality (29). COVID-19 vaccines are currently administered based on the evidence of its security and immunogenicity (30). However, identified immunogenicity of the COVID-19 vaccines reported in prior experiments was approximately 70%, indicating that COVID-19 vaccination is not 100% effective (31). In addition, immunogenicity and duration of the efficacy of COVID-19 vaccines has not been fully elucidated. Moreover, the efficacy of COVID-19 vaccines in varying populations, mainly its ability to prevent infection as well as reducing the risk of mortality, has not been fully explored (32). This may be one of the factors that limit men and individuals with inferior health status from accepting COVID-19 vaccination. Therefore, specific demands and concerns on COVID-19 vaccination for these male students and students with inferior health status should be further explored to increase COVID-19 vaccination coverage. Targeted counseling and interventions can then be implemented to improve protection against COVID-19.

### Social Media Use and KAP Toward COVID-19 Vaccination

The findings of the current study showed that the time spent in using social media and the frequency of using different kinds of social media platforms were positively correlated with the level of KAP toward COVID-19 vaccination. This finding was consistent with findings from a previous study that individuals who were frequently exposed to information on COVID-19 on social media were more likely to accept COVID-19 vaccines and would practice protective measures (25). Searching and browsing COVID-19 information on social media may be a key factor in promoting civil KAP toward COVID-19 vaccination and the main avenue for mobilizing individuals to accept shots of COVID-19 vaccines. Li and Liu reported that frequency of using social media was a higher driving factor for practicing better protective practices against COVID-19, compared with time spent on social media (11). This can be attributed to the fact that individuals rely on varying types of social media instead of a specific one. In the present study, official social media and the public social media were most frequently used for searching information about COVID-19 among college students. However, a previous study reported that public social media and aggregated social media were the most commonly used platforms among Chinese residents (11). Misinformation and inferior quality of information exists in aggregated and public social media compared with official social media, which may contribute to conspiracy beliefs regarding COVID-19 resulting in the reduction of practicing protective measures (11). The findings toward the present study showed that the frequency of using official and the public social media was a positive predictor for KAP of COVID-19 vaccination among Chinese college students. On the contrary, frequency of using aggregated social media was a negative predictor for the KAP toward COVID-19 vaccination. These findings were not consistent with findings from a previous study. Li et al. reported that aggregated social media was a significant predictor for acceptance of protective measures against COVID-19. However, the predicting effect of official social media on the practice of protective measures was not statistically significant. The conflicting findings can be attributed to the fact that college students in the present study trusted official social media more when compared with aggregated social media (8, 33).

Therefore, official and public social media may be key platforms for facilitating and mobilizing acceptance of COVID-19 vaccination for Chinese college students. These findings indicate that information on the benefits of COVID-19 vaccination should be incorporated and promoted in these social media platforms. In addition, the relevant stakeholders should ensure that information quality in aggregated social media, such as in Tencent News, Headlines, Wangyi News, and Baidu News is improved. The government should intensify supervision and management of internet misinformation dissemination.

### eHealth Literacy and KAP Toward COVID-19 Vaccination

The findings of the present study showed that eHealth literacy was positively correlated with KAP toward COVID-19 vaccination among Chinese college students. The findings showed that higher level of eHealth literacy played an important role in improving acceptance and practices of COVID-19 vaccination for college students. A self-developed questionnaire on eHealth literacy on COVID-19 (CoV-eHEALS) was used in a study by An et al. The findings showed that the level of eHealth literacy was positively correlated with knowledge on COVID-19 and adherent actions to prevent the spread of the infection. On the contrary, the level of eHealth literacy was negatively correlated with the level of conspiracy beliefs (34). Consistent findings were observed in the present study. This observation is mainly because individuals with higher level of eHealth literacy are more likely to retrieve information from the internet, and can easily understand, evaluate, and apply this information. In addition, this group is more likely to manage health-related issues through more positive copying strategies (35). These may be the reasons for the high KAP toward COVID-19 vaccination. Notably, m-eHEALS used in the present study determined eHealth literacy based on self-perception, information acquisition, and interactive judgment, and was different from the eHealth Literacy Scale comprising 8 items (eHEALS) commonly used in studies. Gao et al. evaluated eHEALS based on COSMIN guidelines, and the findings showed that the translated eHEALS exhibited an unstable content validity among Chinese population (36). On the contrary, the scientific development process of m-eHEALS ensures good construct validity. Thus, it is a suitable scale for the determination of eHealth literacy for the Chinese population. Results of the m-eHEALS scale were highly consistent with the original scale. The findings showed that self-perception and ability of information acquisition were positive predictors for KAP toward COVID-19 vaccination in the present study. However, interactive judgment was not a predictor of KAP toward COVID-19 vaccination. This finding may be because these three domains of eHealth literacy were highly correlated with one another (*r* = 0.697–0.746, *p* < 0.001). The observation can be attributed to the discrepancy between subjective eHealth literacy assessment with objective appraisals of eHealth literacy on understanding, evaluating, and decision-making of pragmatic health information. Kim et al. reported that individuals with superior self-perceived ability of information retrieval and evaluation show lower rate of correctness when exposed to pragmatic questions (37). Consistent with these findings, college students in the current study showed good eHealth literacy; however, relatively low level of interactive judgment may negatively affect health information processing and health-related decision-making. Acceptance of COVID-19 vaccination was thus compromised. Therefore, improving the ability of evaluating information and decision-making can improve acceptance of COVID-19 vaccination. Moreover, more attention should be focused on populations with lower level of eHealth literacy such as the elderly, rural residents, and the less educated to improve coverage of vaccination. In addition, assessment of eHealth literacy can be optimized by the use of subjective questionnaires combined with objective measuring methods.

The current study had limitations. Firstly, the major limitation was that the study was conducted in China. The accessibility of getting outer information would be fairly different from other countries. Official social media plays a heavy role in providing information in China and would be useful to avoid misinformation, but the situation would be applicable only in China. Secondly, this investigation was only conducted at one university at the Changsha city of China. Therefore, the findings reported in the current study may not be representative of all the college students in China, and generalization of the findings is limited. Further studies should conduct a multicenter investigation or a nationwide survey. Thirdly, college students with education level below bachelor degree were not included in the current study. Finally, self-reported eHealth literacy (m-eHEALS) was determined in the current study, which may differ from the objective levels to some extent. Assessment of eHealth literacy can be optimized by combining subjective questionnaires with objective measuring methods.

## Conclusion

The findings of the current study showed that eHealth literacy and the KAP toward COVID-19 vaccination among Chinese college students were in a relatively high level under the present stage of regular prevention and control of COVID-19. Female students, students with superior health status, students who spend longer durations in using social media, and students who frequently used official and public social media and less frequently used aggregated social media presented higher level of KAP toward COVID-19 vaccination. In addition, students with higher self-perception of eHealth literacy and information acquisition of eHealth literacy showed higher level of KAP toward COVID-19 vaccination. Therefore, male students and students with inferior health status should be targeted for counseling and should be educated on the benefits of vaccination against COVID-19. Moreover, as the most popular social media platforms among Chinese college students, official and the public social media should be utilized for disseminating beneficial health information. Further, the level of eHealth literacy, mainly the ability of interactive judgment, should be improved.

## Data Availability Statement

The original contributions presented in the study are included in the article/Supplementary Material, further inquiries can be directed to the corresponding author.

## Ethics Statement

This study was approved by the Ethics Committee of the Third Xiangya Hospital (ID: I 21059). This study was conducted in the principal of anonymity. Participants agreed to fill in the questionnaires and informed consents were signed.

## Author Contributions

NQ designed this study, performed the statistical analysis, drafted the original manuscript and revised the final paper. SS designed the instrument, collected the data and edited the language. YD and ZS participated in revision of the paper and instructed the statistical analysis. GM, XL, and SZ collected the data. AL and ZZ participated in the design of the study, supervised all the process and controlled the quality of this study. ZZ was responsible for the whole project. All authors contributed to the article and approved the submitted version.

## Conflict of Interest

The authors declare that the research was conducted in the absence of any commercial or financial relationships that could be construed as a potential conflict of interest.

## Publisher's Note

All claims expressed in this article are solely those of the authors and do not necessarily represent those of their affiliated organizations, or those of the publisher, the editors and the reviewers. Any product that may be evaluated in this article, or claim that may be made by its manufacturer, is not guaranteed or endorsed by the publisher.

## Abbreviations

KAP: the knowledge, attitudes and practices
WHO: World Health Organization
m-eHEALS: the mobile eHealth Litercay Scale
CoV-eHEALS: eHealth literacy about COVID-19
eHEALS: the eHealth Literacy Scale.
