# Supplementary material for: Social Media Use, eHealth Literacy, Knowledge, Attitudes, and Practices Toward COVID-19 Vaccination Among Chinese College Students in the Phase of Regular Epidemic Prevention and Control: A Cross-Sectional Survey
Source: Front Public Health. 2022 Jan 27;9:754904. doi: 10.3389/fpubh.2021.754904 (PMC8829334; doi:10.3389/fpubh.2021.754904)
Supplement: Supplementary file 1 [file Data_Sheet_1.docx]

Dear students,

Online social media use plays a critical role in health information dissemination and disease prevention since the outbreak of Covid-19. Therefore, this study aims to investigate the knowledge and attitudes to the Covid-19 vaccination among Chinese college students, as well as the pattern of social media use on searching online health information. We sincerely invite you to complete these questionnaires according to the circumstance of yourself. It will take 4~6 minutes to complete these questionnaires. Your responses would be of great value to our research. Thank you so much for your cooperation!

2021.04.28

Do you give consent to participating in this study?

Yes

No

**Part one: Demographic information**

1. Gender
2. Male
3. Female
4. Age: years
5. What do you major in?

A. medical subjects

B. non-medical subjects

1. What’s your identity?
2. Student
3. Teacher
   1. what’s your education level?
4. undergraduate
5. post-graduate
6. Ph.D candidate
7. How was your health status?
8. Very good
9. Pretty good
10. In General level
11. Pretty poor
12. Very poor
13. Have the relatives, friends, or classmates of you ever been infected with the Covid-19?
14. Yes
15. No
16. How was the possibility do you think you might be infected with Covid-19?
17. Impossible
18. Not likely
19. Likely
20. Most likely
21. Certainly

**Part two: questionnaire for social media use (please answer with the. option that most conformed with the pattern of your daily social media use)**

1. How long did you use social media to search for the information about the COVID-19 in the past week? *[can only answer with one option]*

A. Less than 1 hour

B. 1-2 hours

C. 2-3 hours

D. 3-4 hours

E. 4-5 hours

F. More than 5 hours

2. Which social media platform and how often do you use it to get online information. about the COVID-19? [answer with one option that was most conformed with the pattern of your daily social media use]*

|  | Never | 1-2 times a week | 3-4 times a week | 5-6 times a week | Once a day or more often than that |
| --- | --- | --- | --- | --- | --- |
| Official social media (such as “CCTV news” and “The People's Daily”) | ○ | ○ | ○ | ○ | ○ |
| Medical professional social media (such as “Doctor clove” and “micro medicine”) | ○ | ○ | ○ | ○ | ○ |
| The public social media (such as “Wechat”, “MicroBlog”, “Chinese version of Tiktok”) | ○ | ○ | ○ | ○ | ○ |
| Aggregated social media (such as “Tencent News”, “Tou Tiao”, “Wang Yi News”) | ○ | ○ | ○ | ○ | ○ |

**Part three: mobile-eHealth literacy scale (m-HEALS) for evaluating the e-health literacy for college students. Please answer with one option that is fully conformed with the facts. (1~strongly disagree, 2~disagree, 3~not sure, 4~agree, 5~strongly agree).**

|  | Strongly disagree | Disagree | Not sure | Agree | Strongly agree |
| --- | --- | --- | --- | --- | --- |
| 1. The Internet has helped me a lot in obtaining health-related resources | ○ | ○ | ○ | ○ | ○ |
| 2. I know how to take advantage of the internet to address my questions for health-related issues | ○ | ○ | ○ | ○ | ○ |
| 3. I'm full of confidence in making health-related decisions based on online information | ○ | ○ | ○ | ○ | ○ |
| 4. I am capable of searching in the Internet for useful health-related resources | ○ | ○ | ○ | ○ | ○ |
| 5. I know where to get useful health information in the Internet | ○ | ○ | ○ | ○ | ○ |
| 6. Apart from computers, I am proficient in using Internet mobile devices (such as mobile phones) to target health information | ○ | ○ | ○ | ○ | ○ |
| 7. I will actively search for and learn about health-related knowledge and information in the Internet | ○ | ○ | ○ | ○ | ○ |
| 8. I will circumspectly consider whether it is applicable to my specific health situation even if the targeted health information is credible and in high quality | ○ | ○ | ○ | ○ | ○ |
| 9. I can clearly provide doctors with complete information needed for diagnosis through the internet (such as disease and symptom description, history diseases, physiological examination data, etc.) | ○ | ○ | ○ | ○ | ○ |
| 10. I will pay attention to or participate in a health-related forum, QQ group or Wechat group | ○ | ○ | ○ | ○ | ○ |
| 11. I can distinguish between high-quality and low-quality health-related resources on the Internet. | ○ | ○ | ○ | ○ | ○ |
| 12. I am capable of appraising the quality of online health-related resources | ○ | ○ | ○ | ○ | ○ |

**Part four: questionnaire for the knowledge, attitude, and practices of the COVID-19 vaccination (please answer with one option that most conformed with the facts)**

1. **Please tell whether the following statement about COVID-19 vaccination is correct. (answer with true, false, or don’t know)**
   1. All age groups can be vaccinated with COVID-19 vaccines.
2. True
3. False
4. Do not know
   1. Fever and pregnant women are not allowed to take shots of COVID-19 vaccines.
5. True
6. False
7. Do not know
   1. There is no need to take intervals between COVID-19 vaccines shot and other kinds of vaccines shot.
8. True
9. False
10. Do not know
    1. According to the inoculation procedure, those who missed following 2 or 3 doses of COVID-19 vaccines don’t need to start inoculation from the first shot, instead, they should be re-inoculated from the missed dose as soon as possible.
11. True
12. False
13. Do not know
14. **There are several statements about COVID-19 vaccines. Please answer with one option that can fully represent your opinion. (1~strongly disagree, 2~disagree, 3~ not sure, 4~agree, 5~strongly agree).**

|  | Strongly disagree | Disagree | Not sure | Agree | Strongly agree |
| --- | --- | --- | --- | --- | --- |
| 1. Taking COVID-19 vaccination is very important to my health |  | ○ | ○ | ○ | ○ |
| 2. If I accept COVID-19 vaccination, my families, friends and classmates can be protected from the possibilities of infection |  | ○ | ○ | ○ | ○ |
| 3. I think taking COVID-19 vaccination can reduce the risk of infection |  | ○ | ○ | ○ | ○ |
| 4. It is unnecessary to take shots of COVID-19 vaccines because the COVID-19 pandemic has been well contained at present. |  | ○ | ○ | ○ | ○ |
| 5. I will actively respond to government policies and accept the COVID-19 vaccination. | ○ | ○ | ○ | ○ | ○ |
| 6. I will continue to take protection actions to prevent the spread of infection (such as wearing face masks, avoiding gathering in the crowd) after taking shots of COVID-19 vaccines. | ○ | ○ | ○ | ○ | ○ |
| 7. I will take the initiative to search for online health information about the COVID-19 vaccines. | ○ | ○ | ○ | ○ | ○ |
| 8. I will take the initiative to share health information about the COVID-19 vaccines with people around me. | ○ | ○ | ○ | ○ | ○ |

Thank you for your cooperation again~
