# Supplementary material for: Social Media Use, eHealth Literacy, Knowledge, Attitudes, and Practices Toward COVID-19 Vaccination Among Chinese College Students in the Phase of Regular Epidemic Prevention and Control: A Cross-Sectional Survey
Source: Front Public Health. 2022 Jan 27;9:754904. doi: 10.3389/fpubh.2021.754904 (PMC8829334; doi:10.3389/fpubh.2021.754904)
Supplement: Supplementary file 2 [file Table_1.DOCX]

| **Social media types** | **Frequency [ n(%)]** | | | | |
| --- | --- | --- | --- | --- | --- |
|  | **Never** | **1–2 times a week** | **3–4 times a week** | **5–6 times a week** | **Once a day or more often than that** |
| **official social media** | 414(10.94) | 1939(51.23) | 757(20.00) | 318(8.40) | 357(9.43) |
| **medical professional social media** | 2046(54.06) | 1186(31.33) | 284(7.50) | 133(3.51) | 136(3.59) |
| **public social media** | 634(16.75) | 1592(42.06) | 813(21.48) | 367(9.70) | 379(10.01) |
| **aggregated social media** | 1231(32.52) | 1574(41.59) | 560(14.80) | 219(5.79) | 201(5.31) |

**TABLE1 |** Weekly frequencies in using various types of social media platforms for Chinese college students to search for information regarding COVID-19 (N=3785)
